# Landmark Topology Descriptor-Based Place Recognition and Localization under Large View-Point Changes

**DOI:** 10.3390/s23249775

**Published:** 2023-12-12

**Authors:** Guanhong Gao, Zhi Xiong, Yao Zhao, Ling Zhang

**Affiliations:** College of Automation Engineering, Nanjing University of Aeronautics and Astronautics, Nanjing 211106, China; guanhong@nuaa.edu.cn (G.G.); yaozhaonrc29@nuaa.edu.cn (Y.Z.); zhanglingsnowman@nuaa.edu.cn (L.Z.)

**Keywords:** place recognition, graph matching, global localization, heterogeneous robots

## Abstract

Accurate localization between cameras is a prerequisite for a vision-based heterogeneous robot systems task. The core issue is how to accurately perform place recognition from different view-points. Traditional appearance-based methods have a high probability of failure in place recognition and localization under large view-point changes. In recent years, semantic graph matching-based place recognition methods have been proposed to solve the above problem. However, these methods rely on high-precision semantic segmentation results and have a high time complexity in node extraction or graph matching. In addition, methods only utilize the semantic labels of the landmarks themselves to construct graphs and descriptors, making such approaches fail in some challenging scenarios (e.g., scene repetition). In this paper, we propose a graph-matching method based on a novel landmark topology descriptor, which is robust to view-point changes. According to the experiment on real-world data, our algorithm can run in real-time and is approximately four times and three times faster than state-of-the-art algorithms in the graph extraction and matching phases, respectively. In terms of place recognition performance, our algorithm achieves the best place recognition precision at a recall of 0–70% compared with classic appearance-based algorithms and an advanced graph-based algorithm in the scene of significant view-point changes. In terms of positioning accuracy, compared to the traditional appearance-based DBoW2 and NetVLAD algorithms, our method outperforms by 95%, on average, in terms of the mean translation error and 95% in terms of the mean RMSE. Compared to the state-of-the-art SHM algorithm, our method outperforms by 30%, on average, in terms of the mean translation error and 29% in terms of the mean RMSE. In addition, our method outperforms the current state-of-the-art algorithm, even in challenging scenarios where the benchmark algorithms fail.

## 1. Introduction

Heterogeneous robot systems have efficient and robust performance in forest/urban firefighting, natural disaster search and rescue, underwater/space exploration, and safety inspections [1]. Among them, heterogeneous robot systems centered on visual navigation and localization technology have been widely researched and applied in recent years because of low power consumption, affordability, and the compact size of cameras. Autonomous navigation is a crucial capability for the heterogeneous robot systems in many real-world scenarios. During the long-term operation of the robot system, the relative positions of multiple robots will inevitably shift over time, which requires the robot to handle scene changes to perform global localization robustly. Although vision-based localization problems, closely related to place recognition, loop closure detection, and Simultaneous Localization and Mapping (SLAM) have been extensively studied, there are still many open challenges.

Reliable place recognition and global localization between heterogeneous robots are the core technologies of the heterogeneous robot systems. For place recognition, the key lies in describing a particular place in response to various perceptual variations. The classic appearance-based approach of representing images as vectors or matrices is a widely recognized method by which the similarity between images can be efficiently computed, thereby achieving image-based place recognition. In general, classical appearance-based visual methods for describing the observation of a single image can be divided into two main categories [2]. The first class of methods utilizes local features of the image, such as SIFT [3], SURF [4], and ORB [5] to extract descriptors. These descriptors are used to efficiently encode scene information in images by matching them directly or by quantizing bag-of-words models [6] via clustering methods. The second class of methods considers the image as a whole and extracts the global descriptors of the image, after which the previously reached places are recognized by calculating the similarity of the images, e.g., Gist [7], color histogram [8], and HOG [9]. In addition, some CNN-based methods [10,11,12] also show excellent performance, achieving higher accuracy with shorter descriptors, and are successfully applied in the field of place recognition. Although the above methods have been widely verified to have excellent performance, their applications are often limited when facing scenes with large view-point changes. Significant view-point variation causes significant changes in image features, severely affecting the accuracy of vision-based place recognition tasks and the accuracy of global localization. These appearance-based approaches focus on the local features captured at the moment of observation and cannot describe the whole scene within a region with a macroscopic view. In the case of significant changes in the view-point, the local features change significantly, which leads to the failure of these types of methods.

An effective alternative is constructing graphs based on the semantic information extracted from observations. Since semantic graphs do not change with the view-point, place recognition under large view-point changes by means of graph matching has shown remarkable success in recent years. In addition, semantic graphs make place recognition free from the limitation of local information in a single image by assembling semantic information in a region, thus improving the accuracy and robustness of the algorithm. Some semantic graph-based methods [2,13,14] are proposed to improve image-based place recognition and global localization accuracy under different observation view-points. In particular, some methods based on random walk descriptors [15,16,17,18,19] report inspiring results. These methods construct semantic graphs through dense semantic information as graph nodes, in addition to incorporating vertex neighbor information into the descriptors by introducing their topological relations, making such descriptor-based methods more robust. They are able to successfully cope with global localization under a large view-point change. However, there is still room for improvement. On the one hand, the CNN-based semantic information extraction poses a challenge to the time complexity of the proposed algorithm. Dense semantic information extraction with high accuracy is often accompanied by high time complexity. At the same time, dense semantic information also makes the number of nodes in the graph large, reducing the efficiency of subsequent matching. On the other hand, the robustness of descriptors constructed using only vertex semantic information still needs to be improved in some challenging scenarios.

**Figure 1:**
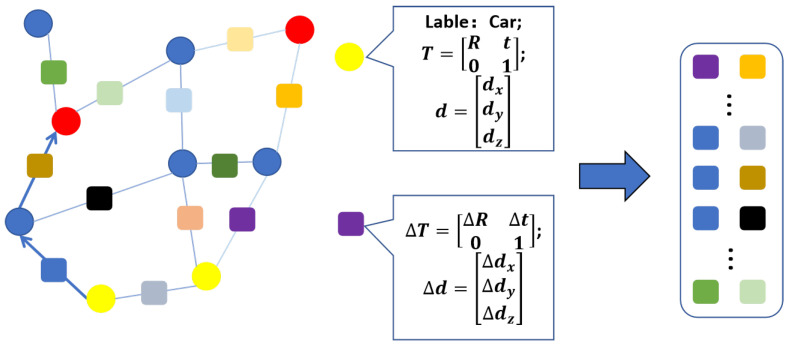
Landmark topology-based descriptor. Left illustrates the undirected semantic graph, and the random walk search path starting from the yellow vertex. The random walk path information is displayed on the right. The circles represent the graph vertices with properties such as semantic label, pose, and size. The squares represent the information recorded by the random walk path, including pose and size changes between connected vertices.

In this paper, we address the challenges discussed above by designing a more reliable and efficient method. Inspired by the approaches [16,19], we employ the graph random walk approach and propose a novel landmark topology descriptor, making the graph descriptor more robust. Based on the new type of descriptor, our approach achieves graph matching and further enables place recognition under significant view-point changes and global localization for heterogeneous robots. Unlike state-of-the-art algorithms that utilize only dense semantic information, our approach enables sparse landmarks to construct topological graphs. Specifically, three-dimensional landmarks that can be detected in the environment are used as graph vertices, thereby effectively simplifying the number of vertices in the graph and the computation time. Moreover, we store the surrounding information by recording the relative transformation relations between connected vertices instead of the information of each vertex only. Hence, we ensure sufficient surrounding information while maintaining a small number of vertices. Furthermore, adding relative relationships makes the descriptors more specific, making our algorithm more robust in challenging environments. Our new descriptor is illustrated in Figure 1. Finally, we implement the global localization of heterogeneous robots based on the graph matching results, then solve the rotation and translation between heterogeneous robot trajectories using the least squares method and evaluate the performance in real image data. From the perspective of its design, implementation, and experimental validation, we make the following contributions in this paper:

- We propose a new random walk descriptor for efficiently placing recognition under a significant view-point change;
- We show that our approach improves the solving speed compared to the advanced real-time graph-based algorithm;
- We present place recognition performance and localization accuracy evaluations compared to traditional appearance-based and state-of-the-art semantic-based methods. Our method outperforms the above methods on the real-world dataset.

The remainder of this paper is organized as follows. In Section 2, we review the previous literature covered by the work. Next, we describe the framework of the proposed system and the detailed algorithmic principles in Section 3. Section 4 shows the experimental results on the KITTI odometry dataset. Finally, we conclude in Section 5.

## 2. Related Work

The field of vision-based heterogeneous robotic systems has received significant attention due to the advantages of vision sensors. In order to meet the global localization needs of deploying visual heterogeneous robot systems in real environments, more and more researchers put attention to place recognition research and proposed various algorithms. These algorithms can be roughly divided into three categories.

### 2.1. Appearance-Based Place Recognition

Appearance-based place recognition is well-studied in computer vision and the SLAM field. A class of approaches uses local or global image features to generate a bag of words that holistically describes the images and measures the similarity between images based on this description. The typical one is FAB-MAP [20], which learns a generative model of place appearance. In addition, Gálvez et al. proposed a novel visual place recognition method DBoW2 [6], which uses the bag of words obtained from the accelerated segment test (FAST feature points [21]) and BRIEF descriptor [22]. The DBoW2 method sped up correspondences for geometrical verification by constructing a vocabulary tree that discretizes a binary descriptor space, achieving a significant real-time performance in place recognition. This method is the most widely used place recognition and localization method for heterogeneous robot systems [23,24], and is also widely applied in several SLAM systems [25,26,27,28,29].

With the development of deep neural networks, CNN models are gradually used for observation scene matching for their excellent recognition accuracy and robustness. Arandjelovic et al. developed NetVLAD [10] based on the vector of local aggregated descriptors (VLAD). This CNN-based method uses GPS information as labels for weakly supervised training, and can generate feature vectors of the global information of images. Compared with traditional methods, the NetVLAD method can achieve higher accuracy with shorter descriptors, effectively reducing data transmission.

Although the above methods are competent in place recognition tasks with similar view-points (e.g., loop closure detection or relocation in SR-SLAM), their performance still needs to be improved in scenarios with significant view-point changes.

### 2.2. Semantics-Based Place Recognition

In addition to generating global descriptors of images, CNN models have been used more in analyzing the environment and providing semantic information in images in recent years of research. Choudhary et al. [30] generated visual words for loop closure detection by YOLO [31] object detection network. Mousavian et al. [32] proposed a method to significantly improve the performance of bag-of-words models by employing a CNN-based image semantic segmentation model to filter out feature points in buildings. Inspired by [32], Tayyab et al. proposed a new method for learning a discriminative holistic image representation, which can accurately segment regions in the image that remain geometrically stable over a long period. In addition, Yu et al. [18] proposed a method to construct RGB-D maps and achieve place recognition through sparse object graphs.

### 2.3. Approaches with Graph Representation

In recent studies, graph-based approaches have received attention and have been studied in the field of place recognition and global localization, mainly because graph structure is invariant to changes in view-point. Graph representation encodes global features (vertices) and their topological relations (edges), reflecting the information from a single observation and the association between multiple observations. Moreover, such methods focus on matching between graphs by finding correspondences between vertices. Place recognition is achieved by graph matching, and global localization is achieved by solving the transformation between graphs.

Among the many algorithms, extracting vertices using image semantics has become mainstream [15,16]. In [2,33], the authors use CNN-based 2D detection algorithms to label objects in images as vertices and associate them using the Hungarian and KM algorithms, respectively. Moreover, ref. [18] extract graph vertices as 3D objects by RGB-D camera to combine high-level 3D semantic information and low-level feature information for place recognition and loop closure detection.

To improve the efficiency and localization performance of the graph representation in the outdoor environment, Gawel et al. proposed a graph descriptor method named X-View [16]. This method employs pixel-level semantic segmentation to extract objects from the images and uses the semantic labels as graph vertices. Place recognition under significant view-point changes is achieved by constructing and associating vertex random walk descriptors. However, multiple accesses between vertices increase the time complexity of the system significantly when the environment is significant. To solve this problem, Guo et al. focused on improving the real-time performance and accuracy of graph-based methods and improved X-View by proposing a semantic histogram-based descriptor (in this paper, we call this method the semantic histogram method and abbreviate it as SHM to avoid a redundancy in the text) [19], which takes odometry data, semantic maps and depth maps as system inputs, extracting dense semantic labels as vertices and constructs un-directed graphs and vertex descriptors. This method uses a pre-arranged histogram form to store the surrounding information, while the graph matching is formulated as a dot-product between two descriptor sets. This more accurate method enables graph matching and global localization operations to run in real-time. However, relying on high-precision dense semantic segmentation and depth estimation for extracting graph vertices limits the further efficiency of such algorithms. Additionally, constructing graph descriptors using only the semantic information of the individual vertex makes some complex scenarios challenging.

## 3. Method

**Figure 2:**
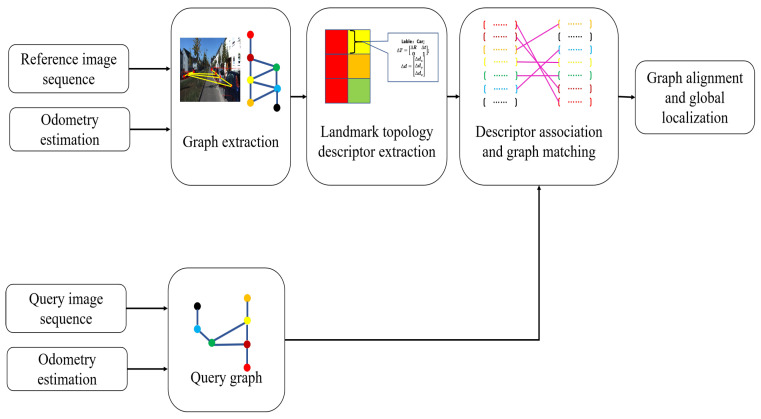
The diagram of our place recognition and global localization system. Our approach takes RGB image stream and odometry data as input. Then, 3D landmark extraction and fusion are performed to build a reference graph. Meanwhile, the same operation is conducted for another robot image sequence to build a query graph. Next, random walk descriptors based on relative relations are obtained for all vertices in the graphs. After that, we correlate the random walk descriptors of two graphs to find the best graph correspondence. Finally, the 6-DoF transformation between the two robot coordinates is estimated based on the calculated matching results between the graphs.

This section introduces our place recognition and global localization system based on landmark topology. The framework of our method is shown in Figure 2. Unlike the state-of-the-art methods, we not only utilize landmark semantic information, but we also extract and add landmark pose and size information into the graph construction. The random walk method is used to construct descriptors of graph vertices and is used for vertex descriptor matching. Based on the matching results, an Object-RANSAC method is proposed to eliminate incorrect node matches. Finally, global localization is implemented by computing the rigid transformation between the two matching graphs using the ICP algorithm.

The system consists of five main modules: landmark extraction, graph extraction, descriptor extraction, graph matching, and global localization. First, our system only inputs two RGB image sequences and visual odometry estimation. To obtain landmark vertices that can be used to construct the to-be topological map, 3D-level object detection is computed for each frame to extract landmarks from the image stream. To achieve this, we use the monocular image-based real-time 3D object detection algorithm SMOKE [34] to achieve end-to-end landmark detection. This CNN-based method provides the semantic label, pose, and size information of the landmarks.The specific construction method of landmark vertices will be introduced in detail in the following subsection. Second, the system generates a reference and a query undirected semantic graphs based on the detected landmarks. Third, vertex descriptors are extracted based on the relative relations between landmarks and their neighbors. In the end, we associate two vertex sets and calculate the 6-DoF transformation matrix between graphs.

In order to ensure that the constructed descriptors are identical and matching, in the descriptor matching stage, we use a combination of calculating similarity scores and eliminating incorrect matches to obtain correct descriptor matching results. First, only graph vertices with the same semantic label are compared. Second, the error between each random walk path of the descriptor pair to be matched is calculated to obtain the similarity of each random walk path of the descriptor to be matched. Based on the number of matching random walk paths, the descriptor similarity score is calculated for the preliminary screening of pairs to be matched, thereby obtaining candidate matching pairs with higher similarity. Furthermore, the Object-RANSAC method was proposed to eliminate erroneous matches in the above candidate matching team, based on the vertex position and attitude errors of the candidate matching pairs after rigid transformation. Finally, the correct descriptor matching result is obtained.

### 3.1. Landmark Detection and Object-Level Vertex Extraction

In order to construct a topological graph that can be matched under different view-points, it is necessary to extract distinctive and view-invariant semantic features as vertices from the observation. In some methods, like [16,19], each instance obtained by image semantic segmentation is used as a graph vertex and participates in subsequent matching, making the number of vertices in the semantic graph larger. As the number of vertices increases, the number of random walk times during the descriptor construction and the number of vertex traversals during the matching process also increase, which significantly increases the time complexity of the algorithm. On the other hand, adding all semantic information to the graph is unnecessary since several landmarks do not have exact locations, such as roads, sky, and grass. Therefore, we pay more attention to observing landmarks like parked vehicles or trucks rather than global semantic features in the images. The above objects are detected in the application of autonomous driving as a wide range of semantic features in the urban environment. Although parked vehicles are identified as potential dynamic targets in many SLAM tasks, in several other studies [2,14,19], the time interval between each matching operation is considered short. Therefore, most parked objects can still be added to the topological graph as important static landmarks, which is obviously reflected in KITTI-Residential [35]. In this way, the number of the vertices in the graph is effectively reduced.

As we reduce the number of vertices in the graph, the neighboring information available for matching is simultaneously reduced. To integrate more information, in addition to the semantic labels, we add the pose and size of the landmarks as vertex attributes into the graph. More importantly, we further compute transformation relations between vertices. The specific application will be described in detail in the subsequent sections. To this end, we employ the end-to-end 3D object detection algorithm for the vertex extraction. For each detected object, we present it as a quadruple:

(1) $$\begin{array}{cc} o_{i} & =\left[\begin{array}{c} l_{i}\\ R_{i}\\ t_{i}\\ d_{i} \end{array}\right]\\ \mathrm{with}\;t_{i} & =\left[\begin{array}{c} t_{x}\\ t_{y}\\ t_{z} \end{array}\right]d_{i}=\left[\begin{array}{c} d_{x}\\ d_{y}\\ d_{z} \end{array}\right] \end{array}$$

Here, $l_{i}$ represents the semantic label of landmarks, $R_{i}$ and $t_{i}=\left[t_{x},t_{y},t_{z}\right]$ represent the pose and location in the local world coordinate, and $d_{i}=\left[d_{x},d_{y},d_{z}\right]$ represents the size of the landmarks. The landmark semantic label determines whether two vertex descriptors need to be matched. Specifically, only graph vertices with the same semantic label are compared. The remaining attributes of the landmarks are used to calculate the relative transformation relations between vertices during the random walk to construct vertex descriptors.

### 3.2. Undirected Graph Generation

After the vertex extraction phase, we employ the undirected graph G = <V,E> to model the topology of all objects in the map, where *V* represents the set of unique vertices and *E* represents the set of the binary edges between elements in *V*. Since there is an error in the object pose detection in each frame, it is necessary to associate the object observed at the current moment with the vertics in the graph. In the graph, vertices extracted from different frames with the same semantic label are merged. We do not just associate the vertices between adjacent frames but compare the objects in the current frame with the vertices that already exist in the graph and fuse the vertices with an euclidean distance that is smaller than the object fusion threshold. Then, the undirected edges are added between any two vertices within a proximity distance, which implicitly expresses the spatial relationship of the connected landmarks. Note that, for unconnected vertices, we further search for potential edges in incremental steps of 5 m based on the proximity distance, which minimizes the occurrence of the isolated vertex while keeping fewer edges in the graph.

### 3.3. Landmark Topology Descriptor Generation

In order to describe each vertex in the above graph structure, it is necessary to record the surrounding information of the vertices by extracting the descriptor of the vertices. Since the space complexity in the graph is simplified, we construct our new random walk descriptor based on the spatial topological information to ensure robustness. Unlike the state-of-the-art algorithms, we construct new random walk descriptors based on spatial topological information, and our descriptor takes two connected vertices instead of a single vertex as one random walk step. In particular, it computes and records the pose and dimensional differences between connected landmarks in each step instead of the semantic label only. Therefore, the neighboring information of the graph can still be maintained. Furthermore, the descriptors implicitly express the pose attributes of the vertices, which makes the descriptor more unique.

Since outdoor detectable landmarks are sparser than indoors, we set the depth *N* of the random walk path to be greater than two to ensure that sufficient surrounding information is included, which means that more than three vertices are traversed at a time. For each landmark vertex, we use the random walk method to traverse its first-level adjacent vertices and second-level adjacent vertices; thereby, the descriptor stores all possible paths starting from that vertex. We call each random walk traversal a path. Since the descriptor in this paper stores the spatial information change values between two connected landmarks rather than the information of each vertex individually, each path can be regarded as a (N−1)-dimensional vector storing the pose change and size change of each walk step, respectively. The information recorded by each component of the path during the random walk process is referred to by descriptor cells. The descriptor construction method is described in Algorithm 1.
**Algorithm 1** Descriptor Extraction**Require:** An undirected graph G=(V,E).**Ensure:** 
Vertex descriptor set *H*.1:**for** each vertex $n_{i}\in G$ **do**2:   Initialize the path descriptor vector $v_{i}$ and the path descriptor matrix $H_{i}$;3:   Traverse all vertices connected with $n_{i}$;4:   Obtain the object state $o_{i}=\left[l_{i},R_{i},t_{i},d_{i}\right]$;5:   **for** each vertex $n_{n}$ connected with $n_{i}$ **do**6:       Compute and record the differential value including the rotation error $\Delta R_{n}$, translation error $\Delta t_{n}$, and 3-DoF size error $\Delta d_{n}$;7:       **for** each vertex $n_{nn}$ connected with $n_{n}$ **do**8:            Compute and record $\Delta R_{nn}$, translation error $\Delta t_{nn}$ and 3-DoF size error $\Delta d_{nn}$;9:       **end for**10:       Complete the path descriptor vector $v_{i}=\left[\begin{array}{c} \Delta R_{n},\Delta t_{n},\Delta d_{n}\;\Delta R_{nn},\Delta t_{nn},\Delta d_{nn} \end{array}\right]$11:   **end for**12:   Add $v_{i}^{T}$ into $H_{i}$13:**end for**14:Add $H_{i}$ into *H*

### 3.4. Vertex Matching

After constructing the reference and the query graphs $G_{a}=\left\{\begin{array}{c} v_{a}^{1},v_{a}^{2},\ldots ,v_{a}^{n} \end{array}\right\}$, $G_{b}=\left\{\begin{array}{c} v_{b}^{1},v_{b}^{2},\ldots ,v_{b}^{m} \end{array}\right\}$ and their vertex descriptors, we associate the vertices in the two graphs by calculating the similarity scores across each corresponding descriptor. The similarity is calculated by matching each candidate descriptor correspondence. Specifically, the similarity is represented by the vertex similarity score, which contains the similarity score for each path in the vertex descriptor. Therefore, we need to evaluate the error between the corresponding descriptor cells in each path vector. The cell error can be computed by the following formulation:

(2) $$\left\{\begin{array}{c} e_{R}={∥\Delta R_{a}-\Delta R_{b}∥}_{F}\\ e_{t}={∥\Delta t_{a}-\Delta t_{b}∥}_{2}\\ e_{d}={∥\Delta d_{a}-\Delta d_{b}∥}_{2} \end{array}\right.$$

(3) $$e_{vertx}=w_{R}e_{R}+w_{t}e_{t}+w_{d}e_{d}$$

Here, $w_{R}$, $w_{t}$, and $w_{d}$ are the weighting parameters. The random walk path can be considered as matched if the corresponding cell error satisfies the following constrain:

(4) $$e_{vertex}<T_{n}$$

It is worth noting that only the descriptor with the same start vertex semantic labels will be compared. In a matching process, the number of matching random walk paths *p* in the two descriptors to be matched reflects the similarity of the two descriptors. The similarity score of the two descriptors after normalization is shown in Equation (5):

(5) $$s=\frac{p}{P}$$

Here, *P* denotes the number of paths of the descriptor random walk. The percentage of matched paths in the descriptor reflects the similarity between the two vertices. A higher score responds to a higher degree of similarity.

However, since landmark objects in outdoor environments may have close shapes, sizes, and arrangements, multiple vertices to be matched will exhibit the same similarity score. Based on the basic idea of RANSAC [36], we propose an Object-RANSAC method to cull incorrectly matched descriptor pairs. The specific procedures are shown in Algorithm 2. We randomly select candidate matching pairs as the benchmark and calculate the rigid transformation matrix between the two graphs. Once $T_{0}$ is initialized, we evaluate all the other candidate matches from the correspondence set and compute the transformed pose errors based on this transformation. The candidate correspondences are considered as correct matches if the transformed pose errors are less than the threshold $T_{R}$.
**Algorithm 2** Best Match Selection**Require:** 
Candidate correspondences set $M_{0}$; Rejection threshold $T_{R}$; correspondence number $N_{0}$**Ensure:** 
Optimal matching *M*.1:Initialize $M_{0}$, *M*;2:Initialize maximum inlier number $I_{max}=0$;3:**for** i=0 to $N_{0}$ **do**4:   Select 1 correspondence $C_{i}=\left\{\begin{array}{cc} v_{a} & v_{b} \end{array}\right\}$5:   $T_{0}^{i}=T_{nb}{T_{N{}_{a}}}^{-1}$6:   **for** The rest candidate correspondences $C_{i}=\left\{\begin{array}{cc} v_{a}^{j} & v_{b}^{j} \end{array}\right\}$ **do**7:     $Error=∥T_{n_{b}}^{j}-T_{0}T_{N_{a}}^{j}{∥}_{F}$8:     **if** $Error<T_{R}$ **then**9:        Add $C_{i}$ into $M_{0}$;10:     **end if**11:   **end for**12:   Compute the inlier number $I=Count(M_{0})$;13:   **if** $I>I_{max}$ **then**14:     $M=M_{0}$;15:     $I_{max}=I$;16:   **end if**17:**end for**

### 3.5. Global Localization

After associating query graph vertices with vertices in the reference graph, we perform pose estimation using the spatial location of the vertices. The localization is achieved by densely aligning the centers of the objects of interest in the query and reference graphs. Our algorithm transforms the query graph to the reference graph coordinate system to achieve a global localization of the two robots. Specifically, we solve the 6-DoF transformation matrix by iterating the closest point algorithm (ICP) [37] and solving it by the SVD [38] method. Therefore, the rotation and translation between the two trajectories are computed by minimizing the sum of the squared error, as follows:

(6) $$E\left(R,t\right)=\frac{1}{A}\sum_{i=0}^{A}∥{p_{a}-Rp_{b}-t}^{2}∥$$

Here, *A* represents the number of correct matching vertices after our culling operation, and $p_{a}$ and $p_{b}$ are the localization of the correct matching vertices in their respective semantic graph coordinate systems.

## 4. Experiments

In this section, we present the experimental setup, the results, and discussions. Our method was evaluated on the real-world KITTI dataset [35]. The experiments start with comparing with benchmarks to evaluate the place recognition performance. Secondly, the localization accuracy and the time complexity of different algorithms were evaluated. Finally, the effect of certain parameters in our algorithm was investigated.

To demonstrate the performance of our landmark topology algorithm, we investigate the contrast to DBoW2 [6], NetVLAD [10], and SHM [19]. The first two methods, as classic appearance-based methods, are widely adopted in vision-based place recognition tasks. The latter is a state-of-the-art real-time graph-based algorithm that can achieve accurate place recognition and localization in scenarios with large view-point changes. Since the data sequence consists of images only, we apply the visual-based ORB-SLAM2 [25] as an odometry estimation. All the methods only took RGB images as input. All CNN algorithms employed are conducted on a platform with an Intel Core i9-12900K (Company: Intel, Santa Clara, CA, USA) and two Nvidia 3090 with 24 GB RAM (Company: Nvidia, Santa Clara, CA, USA). The rest were conducted on an platform with an Intel Core i7-10700K and a Nvidia 3070 with 8 GB RAM.

### 4.1. Dataset and Experimental Setup

**Figure 3:**
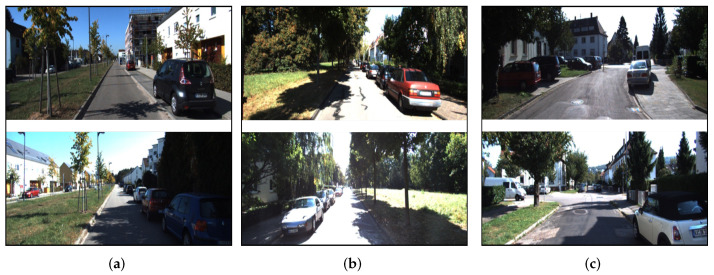
Sample images of different sequences in the experiment. The top and bottom rows represent the forward and backward views, respectively. (**a**) Indicates that the trajectories of the two cars are parallel and go straight towards each other, (**b**) indicates that the trajectories of the two cars coincide with each other and go straight towards each other, (**c**) indicates that the trajectories of the two cars coincide with each other, go straight towards each other and contain curved trajectories.

We implement the experiments on the KITTI odometry dataset. It consists of 22 stereo sequences collected by a car driving in different environments and conditions. Furthermore, we take sequences 06, 08, and 19 to evaluate the proposed method, since these three sequences contain forward-to-backward view trajectories and different trajectory types (straight, turning, and parallel). The sample images are depicted in Figure 3.

**Figure 4:**
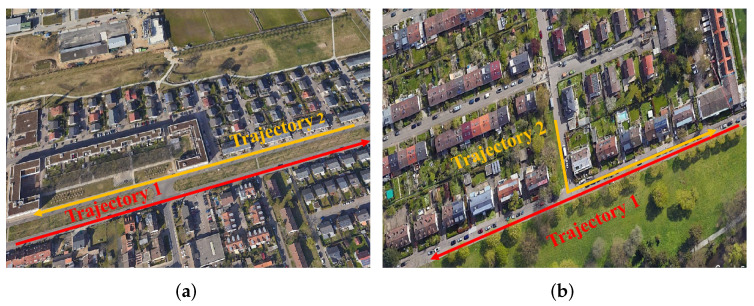
Different types of overlapped trajectories. The left image (**a**) illustrates the challenging overlapped view. The right image (**b**) illustrates the normal overlapped trajectories.

In sequences 08 and 19, two trajectories overlap in a section of the road and pass through the overlap in opposite directions. However, in sequence 06, the two trajectories in opposite directions are parallel without overlapping, which makes the observations of the two cameras more different and poses additional challenges for place recognition and global localization under large view-point changes. The two types of overlapped trajectories are illustrated in Figure 4. The total frame numbers are 253, 546, and 601. The overlap distances are 380 m, 200 m, and 300 m.

Precision-Recall curves (PR-curves) are employed to evaluate the position recognition performance of different algorithms under large view-point changes. In the PR curve, P stands for precision, and R stands for recall. In our experiments, each image represents a place to be recognized and localized. Precision evaluates the proportion of places recognized correctly, and recall denotes the ratio of the recognized places to all places to be recognized. The PR curve takes recall as the horizontal coordinate and precision as the vertical coordinate. Therefore, the PR curve achieves higher precision at higher recall, reflecting better algorithmic location recognition performance. The precision and recall are computed in Equations (7) and (8):

(7) $$Precision=\frac{TP}{TP+FP}$$

(8) $$Recall=\frac{TP}{TP+FN}$$

Here, TP is a true positive, which represents place recognition results that are identified by the algorithm as positive and are actually true. FP is a false negative, which represents place recognition results that are identified by the algorithm as negative but are actually true. FN is a false positive, which represents place recognition results that are identified by the algorithm as positive but are actually false.

For global localization accuracy under large view-point changes, we employ the mean and the root mean square error (RMSE) translation error to evaluate the absolute trajectory error.

### 4.2. Learning-Based Model Setup

In our system, a deep learning-based 3D object detection algorithm SMOKE [34] (ranked at 150/164 by the KITTI official open source leaderboard) is used for landmark detection. This provides the landmark detection results, including the label, pose, and size. As for SHM, we follow the settings in the paper [19] to employ the pixel-level semantic segmentation algorithm Mseg [39] (ranked at 2/6 by the KITTI official open source leaderboard) and the depth estimation algorithm BTS [40] (ranked at 5/19 by the KITTI official open source leaderboard) for landmark detection and localization, respectively. All the CNN algorithms employed were inferred with the pre-trained model on the KITTI dataset and applied with default parameters without additional training.

### 4.3. Place Recognition Performance under Large View-Point Changes

The Precision-Recall curves are generated to evaluate the place recognition performance of different methods. In the experiments, for each matched vertex, we give a positive vote to the image that participated in the construction of that vertex. We change the strictness of the algorithm by varying the matching threshold ($T_{n}$) and the projection threshold ($T_{R}$) to obtain different vertex matching results, and we further count the TP and FN to calculate the Recall. In addition, the localization threshold ($T_{L}$) is employed to evaluate the euclidean distance error between the estimated position $c_{i}$ and the true value $c_{gt}$. We set the estimated position with the distance error less than $T_{L}$ as true, i.e., $∥c_{i}-c_{gt}∥<T_{L}$, so that for the images that the algorithm considers to be positive place recognition results, we count the true position as TP and the false position as FP, thus calculating Precision. To compare with the advanced algorithm SHM, we follow the SHM operation and set $T_{L}$ to 20 m. In addition, we conduct experiments at lower $T_{L}$ to verify the performance under higher criteria. The graph is constructed through a set of all frames, the random walk depth is set to 3, and the landmark fusion threshold is set to 5. Additionally, we initialize the query graph at a zero point with an identity pose. The selection of parameters will be further discussed later. For the appearance-based DBoW2 and NetVLAD methods, we vary the thresholds on the similarity score and the euclidean distance of the feature vector to obtain different precision and recall, respectively.

**Figure 5:**
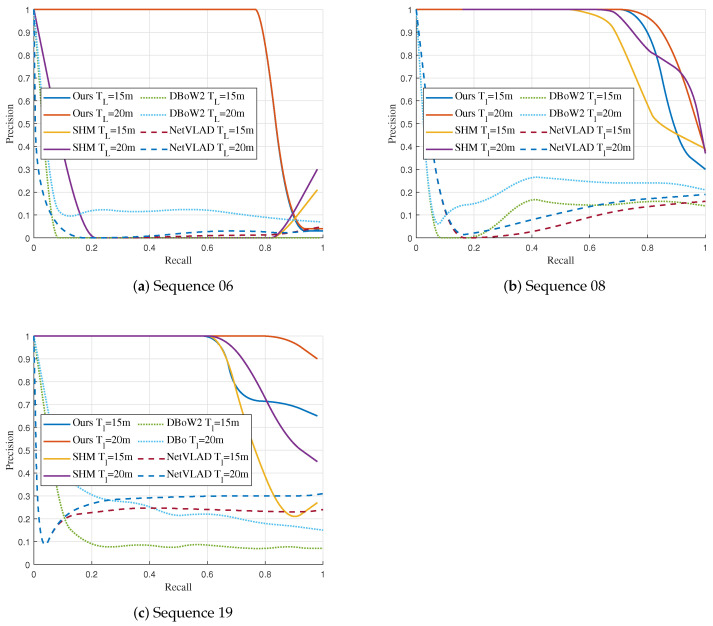
Precision-Recall curves for each sequence.

The results are illustrated in Figure 5. Overall, the appearance-based algorithms DBoW2 and NetVLAD achieved the worst performance. According to our analysis, the reason for these results is these methods only rely on inter-image feature similarity for matching. In this case, when there is a significant change in the observation view-point, the features of the images captured for the same place also change significantly, making the global feature descriptors of the images have large differences and leading to matching failure. Moreover, images taken from different locations are likely to have a high similarity, which also leads to matching failure.

The graph-based approaches have better performance than the appearance-based approaches. In sequences 08 and 19, the graph-based methods can maintain a high accuracy at a higher recall rate. In sequence 08, our method is competitive with SHM. Our method has slightly higher precision in recall below 90% with $T_{L}$ = 20 m and 85% with $T_{L}$ = 15 m. Furthermore, our landmark topology descriptor has better performance compared to the SHM in sequence 19. Following the SHM setting, our landmark topology method has a higher precision when the recall rate is greater than 65%. When decreasing the localization threshold $T_{L}$, our method still maintains high precision and outperforms SHM when the recall rate is greater than 70%.

**Figure 6:**
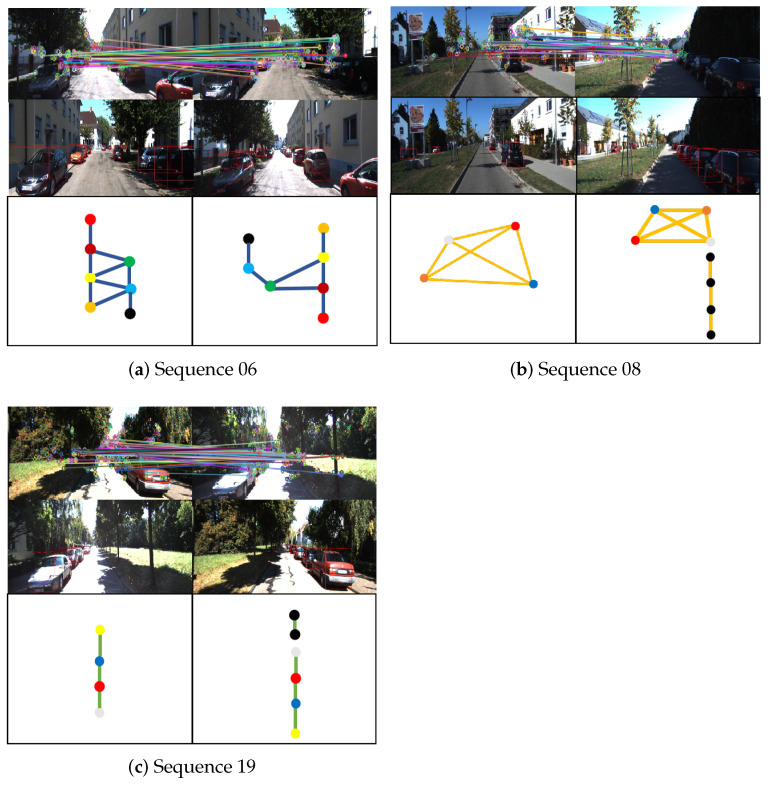
Examples of place recognition for each significant view-point change scenario. The top rows show the failure of traditional key point feature matching under significant view-point changes (e.g., feature points on trees matched with feature points on vehicles or buildings), which clearly demonstrates the difficulty of place recognition in these scenes. The middle rows of images show the landmark vertices extracted in our system. The graphs constructed based on these vertices are shown in the bottom rows, where vertices with the same color and black indicate correspondence and unmatched landmarks, respectively.

It is worth noting that the SHM method fails to succeed in place recognition on sequence 06. We attribute this primarily to two factors. On the one hand, the non-overlapping trajectories are offset in the horizontal direction, which causes some differences in the semantic information around the nodes in the two graphs, as shown in the left image of Figure 4. On the other hand, the semantic information on both sides of the road in the query graph and the database graph has high similarity. This scenario gives non-corresponding vertices high similarity, such as trees on the left in the query graph matching trees on the right in the database graph, leading to errors in matching and rigid transformation solving. Our landmark topology approach constructs descriptors by using transformation relations between vertices regarding poses and dimensions, allowing the descriptors to maintain specificity in this scenario and leading to high matching accuracy. The place recognition and vertex s matching results under large view-point variations are presented in Figure 6.

### 4.4. Time Complexity Analysis

We compared the time complexity with the real-time SHM on KITTI08 and counted the computation time for each module of the algorithms. The time complexity results of different methods are from our own implementations. The result is shown in Table 1. The vertex detection part records the average time for the semantic detection of each image frame, and the other sections record the whole graph processing time. Although the SHM algorithm can achieve real-time operation in the graph matching phase, according to the paper [19], the semantic-only approach relies on a highly accurate semantic segmentation algorithm to achieve a low translation error, which requires a long computation time. In addition, the seed filling algorithm [41] is applied after the semantic map input to segment objects from images, which further increases the graph construction time. Thanks to the lightweight 3D object detection algorithm, we can perform vertex extraction faster and reduce the number of vertices in graphs. This significantly shortens the graph and descriptor construction time, achieving accurate place recognition and global localization without relying on a high-precision vertex extraction result. In addition, our rejection method computes the transformation matrix based on one vertex, which reduces the number of cycles in the RANSAC algorithm, further reducing the time for graph matching.

Overall, our landmark topology method performs better in place recognition in real-world data sequences than the benchmark algorithms, reflected in higher precision and smaller time complexity.

### 4.5. Multi-Cameras Localization Accuracy under Large View-Point Changes

**Figure 7:**
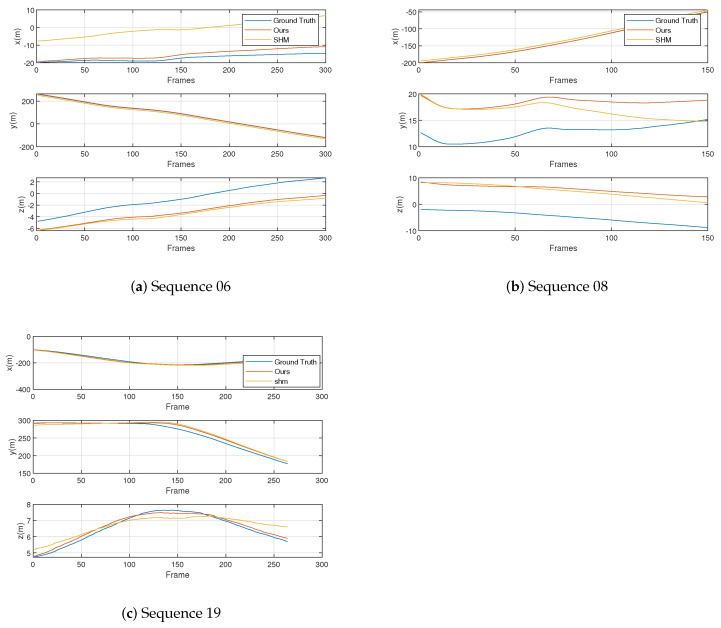
The components of the trajectory on the three axes of different methods.

As in the previous section, we compare our landmark topology method with advanced graph-based and appearance-based methods, respectively. In this section, we evaluate multi-robot localization accuracy on real-world sequences. For the graph-based approach, we compare the RMSE and the mean translation errors of the trajectories. Table 2 shows the localization errors of the four methods. The localization results of the trajectories of different methods in the three coordinate axis directions are shown in Figure 7. Here, the Ground Truth label represents the localization truth value of the trajectories. According to Figure 5 and Table 2, the appearance-based DBoW2 and NetVLAD methods have a large number of false matches and large localization errors in the scenario with large view-point in our experiment, which leads to failure in place recognition. This result makes these two methods unable to complete the trajectory localization in this scenario. Therefore, the DBoW2 and NetVLAD methods are not evaluated in Figure 7.

As shown by the results in Table 2, the appearance-based algorithm is unable to accomplish multi-camera localization under significant view-point changes, with the DBoW2 and NetVLAD algorithms exhibiting the largest localization error. Since the appearance-based method is frame-by-frame matching, we use the best matching inter-frame distance as the localization error. At the same time, according to the results in Figure 5, the appearance-based algorithms are unable to accomplish multi-camera localization under significant view-point changes, resulting in incorrect matching results. Therefore, there is a long distance between the best matching image frames, which makes the localization result based on the matching result wrong and has a large error. Our landmark topology algorithm outperforms the advanced SHM algorithm on all sequences due to higher matching accuracy. Specifically, all the mean and RMSE translation errors are less than 10 m, with a minimum mean error of 2.99 m, RMSE 3.93 m, and a maximum mean error of 6.16 m, RMES 6.18 m. Our algorithm has better performance on both the longest and the shortest trajectories. Compared to the SHM algorithm, the improvement is 29% (mean), 29% (RMSE) and 31% (mean), 28% (RMSE) on sequences 08 and 19, respectively, with the most significant advantage on the longer sequence 19.

For the challenging sequence 06, our algorithm has the absolute advantage in the scenario without overlapped trajectories. Our approach benefits from extending the properties of the vertices and recording relative information between them during random walk operations. Such descriptors contain richer neighbor information and are not significantly affected by the non-overlapped scenario.

### 4.6. Ablation Experiments

This section focuses on investigating how the performance of the algorithm is affected by different parameter settings. Specifically, random walk depth, graph size, object fusion threshold, and initialization options are chosen for the ablation experiments. We evaluate the effect of each parameter by comparing the mean translation error or computation time under different settings. We implement experiments regarding the graph size on KITTI sequence 06, as it contains enough image frames. The rest of the operations were implemented on KITTI sequence 08.

**Figure 8:**
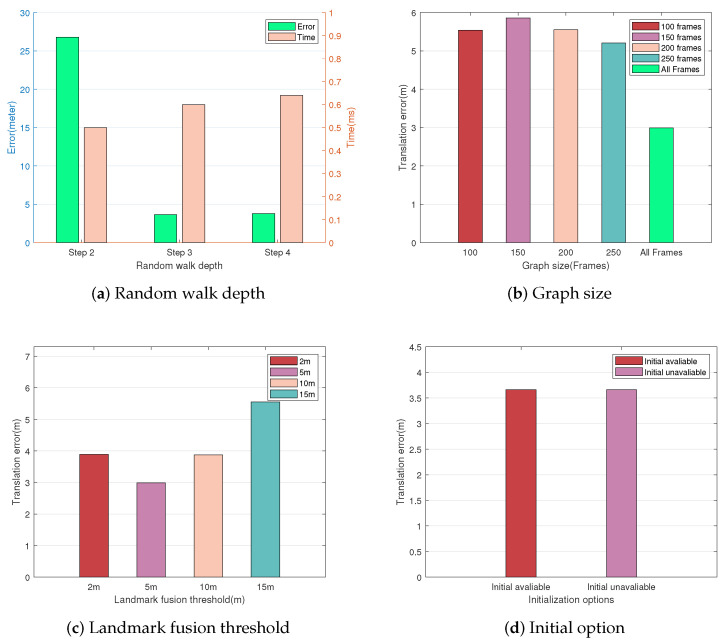
Global localization with different parameters. (**a**) Shows the global localization error and time complexity under different random walk depths. (**b**) Shows the effect of initialization options. (**c**) The effect of graph size. (**d**) Shows the effect of different object fusion thresholds.

We first evaluate the global localization translation error, descriptor construction, and graph matching time under different random walk depths. We chose 2, 3, and 4 steps as the depths to construct our descriptors, respectively. The comparison results are illustrated in Figure 8a. The histogram clearly shows that the translation error is highest at 2 steps. The errors are approximated at depths of 3 and 4 steps, and both errors are less than 5 m. The results indicate that the time complexity of the algorithm tends to increase as the descriptors become complex. However, the localization accuracy does not increase significantly when the random walk depth exceeds 3 steps.

The comparison regarding the effect of graph size is performed in sequence 06 since the sequence contains enough image frames for matching. We generate query graphs of different sizes by varying the number of image frames used to construct sub-graphs to 50, 100, 150, 200, 250, and all frames.

When the graph size is 50 frames, it is too small to extract descriptors with sufficient neighbor information, leading to matching failure. Further analysis from Figure 8b shows that using more image frames for graph construction leads to lower translation errors. Using all frames for descriptor matching performs the best results, with a reduction in translation error up to about 47% compared to other options.

Moreover, Figure 8c shows the effect of different object fusion thresholds on localization accuracy. The fusion threshold determines the data association between adjacent frames. The localization accuracy with the threshold set to 5 is higher than the other configuration. Note that although the more relaxed threshold reduces the number of vertices in the graph, it does not significantly help optimize the computation time. Instead, it causes the number of unique descriptors to be reduced, thus decreasing the matching success rate. Considering the computation time and localization accuracy, we choose two steps of depth, all frames, and the 5 m fusion threshold to build the graphs in our system.

Finally, we evaluate the effect of initialization options on localization accuracy under the same sequence, and the results are shown in Figure 8d. We initialize the query trajectory poses at zero (initial poses are not available) and ORB-SLAM2 estimation (initial poses are available), respectively. The results show that the position estimation has the same translation error under different options, which indicates that our algorithm is robust to initial pose changes.

## 5. Conclusions

In this paper, we propose a new method for constructing random walk descriptors that exploits semantic information, 3D topology, and landmark relative transformation relations for place recognition and global localization under large view-point changes between different robots. Benefitting from the application of graph structure, our method is robust to view-point changes. Our method uses 3D landmarks instead of dense image semantic information for graph construction, effectively simplifying the number of graph vertices as well as the computation time. In addition, 3D landmarks have more accurate spatial coordinates than instances of semantic segmentation, making localization based on this vertex more accurate. In the descriptor extraction stage, we store surrounding information by recording the relative transformation relationship between connected vertices, which enables our method to ensure sufficient surrounding information while maintaining a small number of vertices. At the same time, compared with descriptors that only use a single semantic information, our descriptors contain more attributes, making vertex matching more accurate and further improving localization accuracy. We evaluate our approach under real-world datasets from the public. These data sequences contain trajectories of different lengths with significant view-point changes in opposite directions. Experiment results show that our landmark topology descriptor exhibits high accuracy and robustness in the cases where traditional appearance-based algorithms are severely compromised. Our method has significant advantages over the state-of-the-art graph-based method, especially when there is a non-overlapping trajectories scenario.

In this paper, we assume that all landmark vertices are static and contribute equally to place recognition. Therefore, we will continue our research in place recognition and localization by investigating weight in our topology graphs to treat static and dynamic landmark vertices differently. In addition, we will continue our work and build a dataset focusing on vision-based place recognition under large view-point changes.

## Figures and Tables

**Table 1:** Time cost for each Component.

	Ours	SHM
Vertex Detection	0.02 s	5.45 s
Graph Extraction	2.28 s	10.59 s
Descriptor Extraction	0.25 ms	4.5 ms
Graph Matching	3.7 ms	7.9 ms
Pose Estimation	0.68 ms	0.66 ms

**Table 2:** Global Localization error for different methods on KITTI dataset.

	DBoW2		NetVLAD		SHM		Ours	
Sequence	RMSE [m]	Mean [m]	RMSE [m]	Mean [m]	RMSE [m]	Mean [m]	RMSE [m]	Mean [m]
Seq. 06	310.15	276.35	211.78	188.32	20.29	20.16	**3.93**	**2.99**
Seq. 08	87.28	74.12	46.04	39.74	8.70	8.68	**6.18**	**6.16**
Seq. 19	139.88	108.83	62.2	48.14	8.45	8.10	**6.03**	**5.55**

## Data Availability

No new data were created or analyzed in this study. Data sharing is not applicable to this article.
